# Fate and Biological Activity of the Antimicrobial Lasso Peptide Microcin J25 Under Gastrointestinal Tract Conditions

**DOI:** 10.3389/fmicb.2018.01764

**Published:** 2018-08-03

**Authors:** Sabrine Naimi, Séverine Zirah, Riadh Hammami, Benoît Fernandez, Sylvie Rebuffat, Ismail Fliss

**Affiliations:** ^1^STELA Dairy Research Center, Institute of Nutrition and Functional Foods, Université Laval, Québec, QC, Canada; ^2^Laboratoire Molécules de Communication et Adaptation des Microorganismes, Muséum National d’Histoire Naturelle, Centre National de la Recherche Scientifique, Sorbonne Universités, Paris, France

**Keywords:** microcin J25, lasso peptide, GI TIM-1 model, degradome, duodenum, pancreatic enzymes

## Abstract

The bacteriocin microcin J25 (MccJ25) inhibits the growth of Gram-negative pathogens including *Salmonella* and *Shigella* species, and *Escherichia coli*. This 21-amino acid peptide has remarkable stability to heat and extreme pH values and resistance to many proteases, thanks to a characteristic lasso structure. In this study, we used the dynamic simulator TIM-1 as gastro-intestinal tract model to evaluate the stability and antibacterial activity of MccJ25 during passage through the proximal portion of the human gastrointestinal tract. MccJ25 concentration was measured in the different simulator sections by HPLC, and inhibition of *Salmonella enterica* serotype Enteritidis was evaluated using qualitative and quantitative assays. LC-MS/MS analysis and subsequent molecular networking analysis on the Global Natural Product Social Molecular Networking platform (GNPS) and analysis of the peptide degradation in the presence of proteolytic enzymes mimicking the gastro-intestinal conditions permitted to delineate the fate of MccJ25 through identification of the main degradation products. MccJ25 was relatively stable under gastric conditions, but degraded rapidly in the compartment mimicking the duodenum, notably in the presence of pancreatin. Among pancreatin components, elastase I appeared primarily responsible for MccJ25 breakdown, while α-chymotrypsin was less efficient.

## Introduction

Antibiotics have been used for nearly a century in human and veterinary medicine and for therapeutic or growth-promoting purposes in livestock production (Gustafson and Bowen, 1997; Gyles, 2008). They remain the most widely used chemicals for treating infections. Their use in animal feed began to grow steadily in the early 1950s, when it was observed that keeping animals free of bacterial infections increased livestock productivity considerably and thus reduced production costs (Viola and DeVincent, 2006). However, overuse of antibiotics with structural features similar to those used in human medicine led to the emergence of pathogens with resistance to at least one antibiotic used to treat infections (Reardon, 2014). This worrying development led the European Union to declare a total ban on antibiotics as growth promoters in animal feeds since 2006 (Castanon, 2007). The first industries affected by the ban were hog and poultry production, the principal users of antibiotics as feed additives. It is clear that a satisfactory alternative to antibiotics would be welcome in these industries. Among the alternatives being proposed are natural antimicrobial peptides produced by bacteria, essentially lactic acid bacteria, termed bacteriocins (Ennahar et al., 2000). The production of bacteriocins by bacterial strains is well documented in the literature (Hammami et al., 2013). However, very few bacteriocins have been approved by regulatory agencies for use in food, medical or veterinary products, despite the increasing urgency of the demand for such compounds.

A bacteriocin is most often defined as a peptide of molecular mass mostly <10,000 Da that is inhibitory to species closely related to the producer species. Bacteriocins differ from antibiotics in being synthesized by translation of an mRNA transcript, having generally a narrow spectrum of activity and being effective at concentrations as low as nanomolar (Nes, 2011). Some bacteriocins may undergo post-translational modifications and as such belong to the family of ribosomally-synthesized and post-translationally-modified peptides (RiPPs) (Arnison et al., 2013) Their potential as antimicrobials, especially in food matrices such as dairy, meat and plant products, has been evaluated repeatedly (Cleveland et al., 2001). However, few studies have focused on their potential as inhibitors of enteric pathogens in animals or humans. The stability of bacteriocins in the gastrointestinal tract is uncertain, and their resistance to acid, bile and gastric and pancreatic enzymes is a current research topic. In a previous study, we showed that the class IIa bacteriocin pediocin PA-1 was sensitive to gastrointestinal conditions, losing completely its antibacterial activity in the small intestine (Kheadr et al., 2010).

**FIGURE 1:**
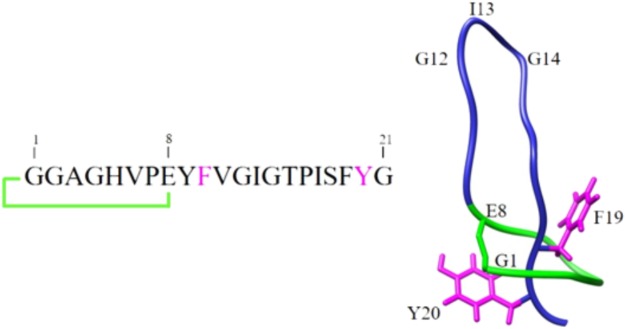
Schematic representation of the primary and three-dimensional structures of MccJ25.

In this work, we examined the potential of microcin J25 (MccJ25), a well-studied bacteriocin produced by the Gram-negative species *Escherichia coli* and of high interest as a potential alternative to conventional antibiotics, because of its potent inhibitory activity against pathogens belonging to the genera *Escherichia, Salmonella*, and *Shigella* (Salomon and Farías, 1992; Sable et al., 2000; Rintoul et al., 2001; Vincent and Morero, 2009). Interestingly its activity is maintained in complex matrices and in a mouse model of infection (Lopez et al., 2007). MccJ25 is produced by *Escherichia coli* AY25 as a translated peptide precursor, which undergoes post-translational modification ensured by two dedicated enzymes (Duquesne et al., 2007; Yan et al., 2012). The process leads to the 21 amino-acid mature peptide (**Figure 1**), which encompasses an N-terminal macrolactam ring of eight residues, through which the C-terminal tail threads and is tightly maintained by steric hindrance of two aromatic bulky side chains (Phe19 and Tyr20) (Bayro et al., 2003; Rosengren et al., 2003; Wilson et al., 2003). This confers to the peptide a unique [1]rotaxane topology known as the lasso structure. This peculiar highly rigid structure has been shown to confer to MccJ25 remarkable stability to high temperatures and extreme pHs (Salomon and Farías, 1992; Blond et al., 2001) and to make the molecule highly resistant to many proteases (Blond et al., 1999; Rosengren et al., 2004). Such features are important if this bacteriocin is to be considered for use as a natural alternative to antibiotics. However, although its properties hold promise, there are no reports on its stability and inhibitory activity under the human GI tract conditions.

The goal of the present study was therefore to evaluate the stability and the inhibitory activity of MccJ25 against *Salmonella enterica* serotype Enteritidis in the upper portion of the human GI tract using a dynamic simulator (TIM-1), as gastrointestinal (GI) tract model.

## Materials and Methods

### Bacterial Strains and Growth Conditions

MccJ25 was produced by a clone of *E. coli* MC4100 carrying a mutated pTUC202 plasmid that confers chloramphenicol resistance (Solbiati et al., 1999). *S. enterica* subsp. *enterica* ser. Enteritidis (Ducasse et al., 2012) was used as a sensitive strain for antibacterial activity assays. Both bacterial strains were obtained from Prof. Sylvie Rebuffat (Muséum national d’Histoire naturelle, MCAM laboratory, Paris, France). As described by Ducasse et al. (2012), *E. coli* MC4100 harboring the pTUC202 plasmid was cultured overnight at 37°C in Luria–Bertani (LB) broth (Difco, Sparks, MD, United States) supplemented with 34 μg/mL chloramphenicol under aerobic condition while *S. enterica* ser. Enteritidis was cultured overnight at 37°C in LB broth under aerobic condition.

### Enzymes and Chemicals

Pancreatin from porcine pancreas, pepsin from porcine gastric mucosa (EC 3.4.23.1), trypsin (EC 3.4.21.4), elastase type IIA from porcine pancreas (EC 3.4.21.36), and α-chymotrypsin from bovine pancreas (EC 3.4.21.1) were purchased from Sigma-Aldrich. Lipase from *Rhizopus oryzae* (EC 3.1.1.3) was from Amano Enzymes Inc. (Nagoya, Japan). All chemicals (HPLC solvents and salts for bacteriological media) used in the study were of high analytical grade.

### Production and Purification of MccJ25

Production and purification of MccJ25 was adapted from Blond et al. (1999) and Rebuffat et al. (2004). Briefly, 3 L of minimal medium (M63) containing KH_2_PO_4_ (3 g/L), K_2_HPO_4_ (7 g/L), (NH_4_)_2_HPO_4_ (2 g/L), and casamino acids (1 g/L) were supplemented after autoclaving with MgSO_4_ 1 mL/L, glucose 10 mL/L, and thiamine 1 mL/L, added as 0.2 μm-filtered solutions (respectively, 20%, 20%, and 1 g/L) and inoculated with *E. coli* MC4100 pTUC202 culture (2% v/v) grown in LB broth. Overnight culture grown at 37°C with rotary shaking at 250 rpm was centrifugated at 8,000 ×*g* for 20 min at 4°C. MccJ25 was isolated from the culture supernatant by solid-phase extraction using a Sep-Pak C18 35 cc cartridge (Waters) washed previously with 200 mL of methanol and 200 mL of ultra-pure water. The bacteriocin was eluted with acetonitrile/water (30% v/v) containing 0.1% HCl. Acetonitrile was eliminated using a rotary evaporator R-215 (Büchi Labortechnik AG, Flawil, Switzerland) before MccJ25 was purified by reverse-phase high-performance liquid chromatography (RP-HPLC, Beckman Coulter System Gold Preparative HPLC system, Mississauga, ON, Canada) on a preparative C18 column (Luna 10 μm, 250 mm × 21.10 mm, Phenomenex, CA, United States) at a flow rate of 10 mL/min using a 25–100% linear gradient of filtered acetonitrile/5 mM HCl with absorbance measurement at 214 nm. The purified MccJ25 eluted as a single peak at 47.69% acetonitrile (Amp: 1.8 AU, 18 min) was lyophilized and stored at -20°C.

### Dynamic Simulator of the GI Tract (TIM-1)

A TIM-1 apparatus (TNO Nutrition and Food Research Institute, Zeist, Netherlands, described previously by Minekus et al. (1995) was used to study the behavior of MccJ25 during passage through the stomach and small intestine. This apparatus is composed of four compartments that simulate the stomach, duodenum, jejunum, and ileum. These compartments are interconnected in series by computer-controlled peristaltic valve pumps (Fernandez et al., 2014). Hollow-fiber membrane dialysis units connected to the jejunal and ileal compartments provide simulation of nutrient absorption without loss of MccJ25. The gastric pH initially set at 4.5 was decreased gradually to 2.7 after 40 min, 2.0 after 60 min and 1.8 after 90 min of digestion by injection of HCl (1 mol/L), and subsequently to 1.7, where it was maintained from 120 to 300 min. The pH was adjusted to 6.3, 6.5, and 7.4 in the duodenal, jejunal, and ileal compartments, respectively, by injection of NaHCO_3_ solution (1 mol/L). Simulated gastric secretions containing pepsin (0.19 mg/mL) or lipase (0.25 mg/mL), both in an electrolyte solution (NaCl 6.2 g/L, KCl 2.2 g/L, CaCl_2_ 0.3 g/L, NaHCO_3_ 1.5 g/L), were delivered into the gastric compartment at a flow rate of 0.25 mL/min. Simulated duodenal secretions consisting of solutions of 8% pancreatin (pancreatin from porcine pancreas 4 × USP, Sigma-Aldrich), 4% porcine bile (porcine bile extract, Sigma-Aldrich) and an electrolyte solution (NaCl 5.0 g/L, KCl 0.60 g/L, CaCl_2_ 0.30 g/L, pH 7.0) were injected at 0.25, 0.5, and 0.25 mL/min, respectively. The total injected volumes were logged. A 2 mg/mL trypsin solution (EC 3.4.21.4 from bovine pancreas, Sigma-Aldrich) was also injected into the duodenal compartment. The initial jejunal and ileal contents consisted of the electrolyte solution at pH 7.0. The fast transit protocol, digestion of a liquid, low-calorie feed condition and adult conditions were applied for all experiments.

The simulated digestion was performed in duplicate. 310 mL of a sterile 0.1 mg/mL MccJ25 solution were injected into the gastric compartment, and aliquots of 7 mL were collected therefrom in duplicate at 0, 30, and 60 min, from the duodenal compartment at 30, 60, and 120 min and from the jejunal and ileal compartments at 60, 120, and 240 min. Each sample was heat-treated at 70°C for 10 min to inactivate all enzymes and then centrifugated at 8300 ×*g* for 10 min at 4°C. The supernatant was loaded onto a Sep-Pak C18 35 cc cartridge (Waters) washed beforehand with 200 mL of methanol, 200 mL of pure acetonitrile and 200 mL of 0.1% formic acid in ultra-pure water. MccJ25 was eluted with 100 mL of 0.1% formic acid solution, and a 1/1 (v/v). blend of filtered acetonitrile and 0.1% formic acid. The eluted fraction containing MccJ25 was concentrated using a rotary evaporator R-215 (Büchi Labortechnik AG, Flawil, Switzerland), lyophilized, re-dissolved in 600 μL of ultra-pure water supplemented with 0.1% acetonitrile.

### Quantification of MccJ25 by Reverse-Phase HPLC

MccJ25 was quantified by reverse-phase HPLC using an analytical C18 column (Aeris™ 3.6 μm, PEPTIDE XB-C18, 250 × 4.6 mm, Phenomenex, CA, United States). A linear standard curve was generated by injecting stock solutions of MccJ25 known quantities (100, 50, 20, 10, 5, 2, 1, 0.5, 0.25, 0.1, 0.05, and 0.025 μg) into the HPLC column at a flow rate of 1 mL/min and a gradient of 100% solvent A (ultra-pure water/0.1% trifluoroacetic acid) and 0% solvent B (acetonitrile/0.1% TFA) to 50% solvent A and 50% solvent B and measuring the absorbance at 230 and 280 nm. The area of the peak corresponding to MccJ25 was calculated by integration using the HPLC software and the standard curve was obtained from the area for each known concentration. Samples collected from TIM-1 compartments and processed as described above were then injected on the HPLC column to monitor the MccJ25 concentrations.

### Static Models Simulating Gastric and Duodenal Digestions

A 50 mL volume of the gastric solution described above was adjusted to pH 1.7. using 1 mol/L HCl. Duodenal solution (35 mL) consisted of 8% pancreatin, 4% porcine bile solutions and 2 mg/mL trypsin solution in intestinal electrolyte solution adjusted to pH 6.3 using 1 mol/L NaHCO_3_. To both solutions, MccJ25 was added to obtain a final concentration of 0.1 mg/mL. They were then shaken at 150 rpm at 37°C. Aliquots of 5 mL were collected in duplicate at 0, 30, and 60 min from the gastric mixture and at 30, 60, and 120 min from the duodenal mixture for MccJ25 quantification and antibacterial activity assays. Each sample was heated at 70°C for 10 min and centrifugated at 8,300 ×*g* for 10 min at 4°C, and the supernatant was mixed with pure acetone (1:1) and centrifugated at 12,000 ×*g* for 10 min at 4°C. The pellet was washed twice with acetone and finally dissolved in ultra-pure water and concentrated using a Speed-Vac concentrator (Model SC110A, Savant Instruments Inc., Farmingdale, NY, United States) to a final volume of 1 mL for MccJ25 determination using the reverse-phase HPLC method described above.

The duodenal experiment was repeated with pancreatin, trypsin, elastase and α-chymotrypsin, each used alone and at a final concentration of 8 mg/mL in the intestinal electrolyte solution. Aliquots of 3 mL were collected in duplicate and analyzed as described above.

### Antibacterial Activity Assays

Inhibitory activity of MccJ25 against *S. enterica* ser. Enteritidis (Ducasse et al., 2012) was tested first using the critical dilution micro-method adapted from Turcotte et al. (2004). In a sterile flat-bottom 96-well polystyrene micro-plate (Falcon; Becton Dickinson, Franklin Lakes, NJ, United States), 125 μL of sample were diluted in twofold series in LB medium, and 50 μL of an overnight LB culture of *S. enterica* ser. Enteritidis diluted 1,000-fold in fresh LB medium were then added to each well. The micro-plate was incubated at 37°C for 18 h. MccJ25 activity was expressed as arbitrary units (AU)/mL using the formula 2^n^ (1,000/125) where 2 is the dilution factor, n is the number of wells showing inhibition of *S. enterica* ser. Enteritidis (absorbance <0.1) and 125 is the volume in μL of the tested fraction.

The inhibitory activity was evaluated qualitatively using the agar well diffusion method described previously by Tagg et al. (1976). Sterile LB (25 mL) containing 0.75% (w/v) agar was seeded with 150 μL of an overnight culture of *S. enterica* ser. Enteritidis and poured into a sterile Petri dish. Wells (7 mm diameter) were cut in the solidified agar using the open end of a sterile 5 mL pipette and filled with 80 μL of tested sample. Plates were incubated at 37°C for 18 h, and the diameter of the zone of inhibition was measured.

### Analysis of MccJ25 Degradation Products by LC-MS/MS

Samples collected from all experiments were analyzed by LC-MS/MS on an ultra-high-performance LC system (Ultimate 3000 RSLC, Thermo Scientific) connected to a high-resolution electrospray ionization – quadrupole – time of flight (ESI-Q-TOF) mass spectrometer (Maxis II ETD, Bruker Daltonics). Separation was achieved on an Acclaim RSLC Polar Advantage II column (2.2 μm, 2.1 × 100 mm, Thermo Scientific) at a flow rate of 300 μL/min, using the following gradient of solvent A (ultra-pure water/0.1% formic acid) and solvent B (HPLC-MS grade acetonitrile/0.08% formic acid) over a total run time of 17.5 min: linear increase from 10 to 60% B for 12 min, linear increase to 100% B for 0.2 min, decrease to 10% B for 0.5 min. The ESI-Q-TOF instrument was externally calibrated before each run using a sodium formate solution consisting of 10 mM sodium hydroxide in isopropanol/0.2% formic acid (1:1, v/v). The MS spectra were acquired in positive ion mode in the mass range *m/z* 60–2,000. The source parameters were as follows: nebulizer gas 35 psi, dry gas 8 L/min, capillary voltage 3,500 V, end plate offset 500 V, temperature 200°C. Automatic MS/MS was carried out using the following set-up: absolute threshold 500 counts, preferred charge states: 1–3, unknown charge states excluded, cycle time 3 s, MS spectra rate: 2 Hz, MS/MS spectra rate: 3 Hz at 5,000 counts increasing to 6 Hz at 50,000 counts or above. MS/MS active exclusion was set after 1 spectrum unless intensity increased fivefold. Collision energy was automatically calculated from *m/z* and charge states. The LC-MS/MS data were treated with Data Analysis 4.3 (Bruker Daltonics).

### Molecular Networking

The high resolution LC-MS/MS data were converted into mgf files using Data Analysis 4.3 (Bruker Daltonics) and subjected to the online GNPS workflow^1^ (Yang et al., 2013; Wang et al., 2016), using the following set-up: parent ion mass tolerance of 0.05 Da, fragment ion mass tolerance 0.05 Da, cluster minimal size 3, minimum matched peaks 4, and minimum cosine similarity score 0.4. Resulting networks were visualized using Cytoscape 3.5.1.

### Nomenclature

To avoid any ambiguity, the rotaxane species generated by hydrolysis of MccJ25 were denoted using the nomenclature previously proposed by our group (Ducasse et al., 2012). They are written {Ax-By/Cz-Dt} where A, B, C, D represent the one-letter amino acid of the extremities of the two associated peptides and x, y, z, t indicate their respective positions in the sequence. The [2]rotaxane product ions generated by MS/MS were termed using the [(b_r_)^∗^(y_s_)] nomenclature previously introduced (Zirah et al., 2011), where r and s indicate the number of amino acids in the non-covalently associated b- and y-type product ions.

## Results

### Stability and Antibacterial Activity of MccJ25 in the Upper Portion of the Human GI Tract

**FIGURE 2:**
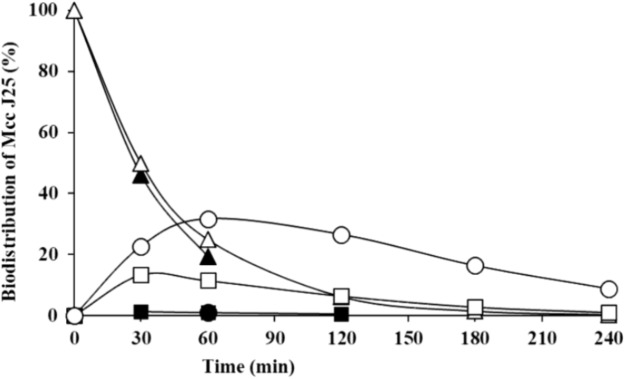
Theoretical and actual distributions of MccJ25 (black: measured values; white: software-calculated theoretical values) in the three compartments of the TIM-1 simulator of the human GI tract over time (triangles, gastric compartment; squares, duodenum; circles, jejunum). Data are means of two independent repetition experiments.

**FIGURE 3:**
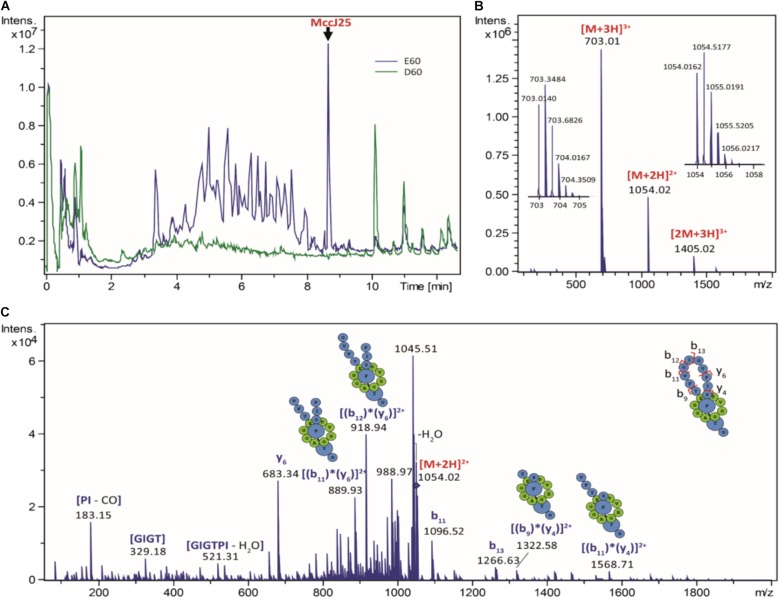
Digestion of MccJ25 in the TIM-1 model. **(A)** Total ion chromatograms generated by LC-MS/MS after 60 min digestion of MccJ25 in the TIM-1 stomach (E60, in blue) and duodenum (D60, in green) sections. **(B)** Positive ion ESI-MS spectrum of MccJ25 detected after digestion in the TIM-1 stomach section (retention time 8.7 min). **(C)** MS/MS spectrum of the [M+2H]^2+^ ion of MccJ25 *(m/z* 1054.02, collision voltage 40 V) showing the typical rotaxane fragment ions diagnostic of the lasso structure.

The stability and antibacterial activity of MccJ25 were first evaluated using a dynamic simulator of the upper GI tract (TIM-1 system). MccJ25 stability was determined using reverse-phase HPLC. **Figure 2** shows the theoretical and actual distributions of MccJ25 in the three TIM-1 compartments over time. In the stomach, the two profiles did not differ markedly, indicating that MccJ25 tolerated gastric conditions. In the duodenum compartment, however, the experimental concentration was significantly lower than the theoretical level, suggesting that the microcin was degraded to a considerable extent at this stage. Samples collected from the TIM-1 model were also tested for their antimicrobial activity against *S. enterica* ser. Enteritidis using the micro-titration assay. As shown in **Table 1**, 1406.2 AU of MccJ25 inhibitory activity were detected per mL of stomach contents at 0 min. This activity decreased after 30 and 60 min, which is likely due to the dynamic nature and function of the TIM-1 system (Minekus et al., 1995). However, the drop to 58.1 AU/mL observed at 30 min in the duodenum and later in this compartment, as well as in the jejunum confirmed the concentration measurements. These results were also confirmed by LC-MS/MS as shown in **Figure 3**. MccJ25 was eluted at 8.7 min and showed doubly charged [M+2H]^2+^ and triply charged [M+3H]^3+^ ions at *m/z* 1054.02 and 703.01, respectively. Its MS/MS spectrum showed the typical rotaxane fragment ions diagnostic of the lasso structure (Zirah et al., 2011; Jeanne Dit Fouque et al., 2018). These fragment ions result from multiple cleavages within the Y9-I18 loop region, which generate b- and y-type product ions associated through steric hindrance provided by the side chains of F19 and Y20. The MccJ25 species was detected as an intense peak in the stomach compartment but was absent from the early stages of the duodenum section.

**Table 1:** Inhibitory activity of MccJ25 against *S. enterica* ser.

TIM-1 compartment	Reaction time (min)	Antibacterial activity (AU/mL)
Stomach	0	1406.2
	30	751.6
	60	848.4
Duodenum	30	58.1
	60	67.7
	120	3.8
Jejunum	60	22.3
	120	13.4

*Enteritidis in the TIM-1 compartments.*

In order to further delineate the nature of MccJ25 degradation products, a molecular network was generated from high-resolution LC-MS/MS data using the GNPS workflow. In the TIM-1 stomach compartment, a single degradation product named DP1 was revealed at 2124 Da (**Table 2**, Supplementary Figure S1, and Supplementary Table S1**)**. The + 18 u increment from the molecular mass of MccJ25 indicated a hydrolysis in the Y9-I18 loop of the peptide, yielding a [2]rotaxane entity, as already reported (Ducasse et al., 2012). The amino acid carrying the + 18 u increment, determined from the MS/MS spectra, permitted to locate the hydrolysis site at G14-T15 (Supplementary Figure S2). The dynamic nature of the TIM-1 model hampered the detection of MccJ25 degradation products formed in the duodenum section, due to the dilution effect. Therefore, the stability and inhibitory activity of MccJ25 were further evaluated in a static model that simulates individually gastric and duodenal physicochemical conditions with a fixed total volume.

**Table 2:** Degradation pattern of MccJ25 in the *in vitro* digestive models.

Incubation time of first detection (min)	Degradation product	Mw (Da)	RT (min)	Assignment	Segment cleaved off
0	–	2106.02	8.6	MccJ25	–
**Stomach dynamic model (TIM-1)**					
60	DP1	2124.03	7.2	{G1-G14/T15-G21}	–
**Duodenum static model**					
0	DP2	2124.03	7.0	{G1-Y9/F10-G21}	–
0	DP3	2124.03	7.3	{G1-F10/V11-G21}	–
0	DP4	2124.03	7.4	{G1-I13/G14-G21}	–
60	DP5	2010.95	6.6	{G1-G12/G14-G21}	{I}
60	DP6	1953.93	6.9	{G1-V11/G14-G21}	{GI}
60	DP7	1854.86	6.6	{G1-F10/G14-G21}	{VGI}
60	DP8	1976.96	6.5	{G1-Y9/V11-G21}	{F}
60	DP9	1707.79	5.7	{G1-Y9/G14-G21}	{FVGI}
120	DP10	1877.89	6.2	{G1-Y9/G12-G21}	{FV}
**Pancreatin**					
0	DP2	2124.03	7.1	{G1-Y9/F10-G21}	–
0	DP3	2124.03	7.3	{G1-F10/V11-G21}	–
0	DP7	1854.86	6.7	{G1-F10/G14-G21}	{VGI}
0	DP8	1976.96	6.5	{G1-Y9/V11-G21}	{F}
0	DP9	1707.79	5.8	{G1-Y9/G14-G21}	{FVGI}
0	DP10	1877.89	6.3	{G1-Y9/G12-G21}	{FV}
**Elastase**					
0	DP3	2124.03	7.3	{G1-F10/V11-G21}	–
0	DP4	2124.03	7.4	{G1-I13/G14-G21}	–
0	DP7	1854.86	6.7	{G1-F10/G14-G21}	{VGI}
0	DP8	1976.96	6.5	{G1-Y9/V11-G21}	{F}
0	DP9	1707.79	5.8	{G1-Y9/G14-G21}	{FVGI}
0	DP10	1877.89	6.3	{G1-Y9/G12-G21}	{FV}
**Chymotrysin**					
0	DP2	2124.03	7.1	{G1-Y9/F10-G21}	–
0	DP3	2124.03	7.3	{G1-F10/V11-G21}	–

*Incubation time of first detection (min), molecular weight (Da) and retention times (RT, min) of MccJ25 degradation products.*

**FIGURE 4:**
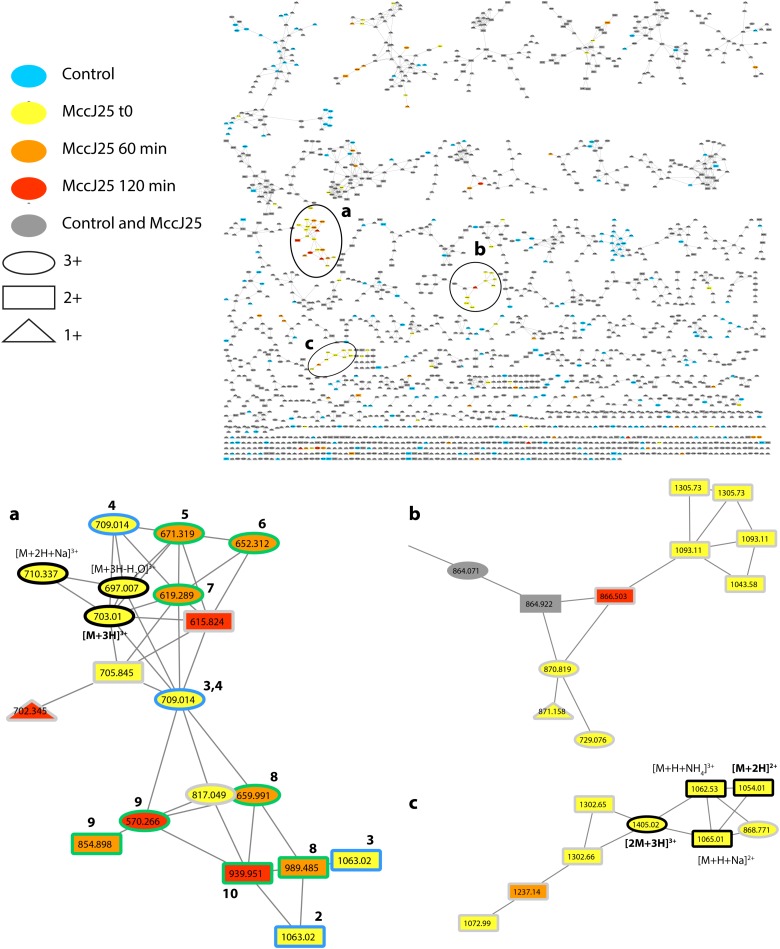
Molecular network showing MccJ25 degradome in the static model of duodenum digestion. The three clusters **(a–c)** corresponding to the degradation compounds derived from MccJ25 are enlarged. The nodes assigned to MccJ25 are bordered in black and annotated. The nodes corresponding to degradation products with one and two hydrolysis are bordered in blue and green, respectively, and annotated with numbers referring to **Table 2**. The nodes, which were not considered to delineate the degradome are bordered in gray. They correspond to compounds with *m/z* values higher than those of MccJ25 or to mixed precursor selection in MS/MS experiments.

A molecular network constructed from the LC-MS/MS data of the duodenal incubations permitted to determine the degradation pattern of MccJ25 in duodenal conditions (**Figure 4**). Analysis of the a, b, and c clusters revealed nine degradation products named DP2 to DP10 (**Table 2** and Supplementary Table S1), which hydrolysis sites were determined from the MS/MS spectra (Supplementary Figures S3–S11), as described above. These degradation products, which differ from the degraded form DP1 identified in the stomach, result from either single or multiple hydrolyses in the Y9-G14 region. The degraded forms DP2 to DP4, detected from t_0_, correspond to hydrolysis of MccJ25 at Y9-F10 (Supplementary Figure S3), F10-V11 (Supplementary Figure S4), and I13-G14 (Supplementary Figure S5) peptide bonds, respectively. The six other products (DP5 to DP10) formed at 60 or 120 min correspond to hydrolysis of MccJ25 at two cleavage sites, leading to the release of a single amino acid or of two to four amino acid peptide segments (Supplementary Figures S6–S11).

Measurement of the inhibitory activity of MccJ25 against *S. enterica* ser. Enteritidis in the static model simulating gastric and duodenal conditions confirmed these results (**Table 3**). The antibacterial activity measured at t_0_ and after 30 and 60 min under gastric conditions was 16,384 arbitrary units per mL. This activity decreased significantly (to 4096 AU/mL) as soon as the beginning of exposure to duodenal conditions, confirming that MccJ25 is degraded instantly in the duodenum. MccJ25 activity increased unexpectedly at 60 min (to 8192 AU/mL) from its level measured at 30 min, then decreased at 120 min to the initial value.

**Table 3:** Inhibitory activity of MccJ25 against *S. enterica* ser.

Condition	Reaction time (min)	Antibacterial activity (AU/mL)
Stomach static model	0	16,384
	30	16,384
	60	16,384
Duodenum static model	0	4,096
	60	8,192
	120	4,096
Positive control	–	32,768
Pancreatin	0	4,096
	120	512
Elastase	0	128
	120	8
Chymotrypsin	0	2,048
	120	1,024

*Enteritidis in the stomach and duodenal static model and in the presence of pancreatin, elastase or chymotrypsin, based on the micro-dilution assay.*

### Stability and Antibacterial Activity of MccJ25 in the Presence of Proteolytic Enzymes

**FIGURE 5:**
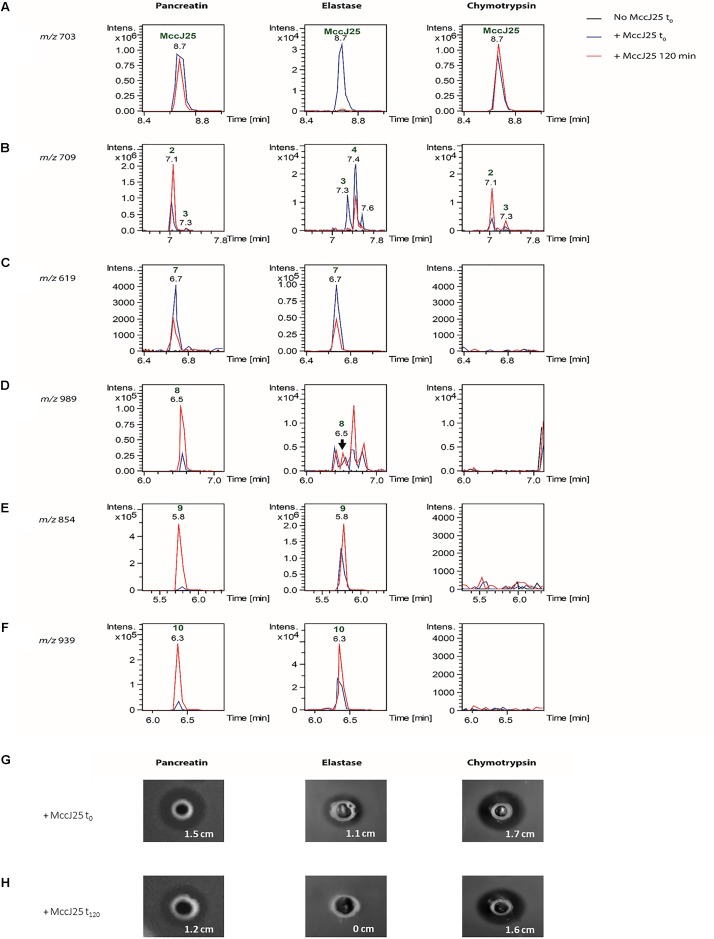
**(A)** Detection of MccJ25 and selected degradation products formed in the presence of duodenal proteases (left: pancreatin, middle: elastase, right: chymotrypsin). Extracted ion chromatograms (*m/z* ± 0.01) of **(A)** *m/z* 703.01 corresponding to the [M+3H]^3+^ species of MccJ25, **(B)** *m/z* 709.02 corresponding to the [M+3H]^3+^ ion of degradation products with a single hydrolysis in the loop region of MccJ25, **(C)** *m/z* 619.29 corresponding to the [M+3H]^3+^ species of compound DP7 {G1-F10/G14-G21}, **(D)** *m/z* 989.49 corresponding to the [M+2H]^2+^ species of compound DP8 {G1-Y9/V11-G21}, **(E)** *m/z* 854.90 corresponding to the [M+2H]^2+^ species of compound DP9 {G1-Y9/G14-G21}, **(F)** *m/z* 939.95 corresponding to the [M+2H]^2+^ species of compound DP10 {G1-Y9/G12-G21}. The peaks are annotated with the compound numbers defined in **Table 2**. **(B)** Inhibitory activity of MccJ25 against *S. enterica* ser. Enteritidis in the presence of pancreatin, elastase and chymotrypsin either upon initial contact (t_0_) or after 120 min of contact (t_120_), based on the agar diffusion assay. **(G)** Inhibitory activity of MccJ25 against *S. enterica* ser. Enteritidis in the presence of pancreatin, elastase and chymotrypsin upon initial contact (t_0_) based on the agar diffusion assay. **(H)** Inhibitory activity of MccJ25 against *S. enterica* ser. Enteritidis in the presence of pancreatin, elastase and chymotrypsin after 120 min of contact (t_120_) based on the agar diffusion assay.

In order to determine which duodenal component is responsible for the breakdown of MccJ25, the effects of pancreatin, trypsin, elastase and chymotrypsin were examined separately in the duodenal solution. The degradation of MccJ25 in these conditions was first monitored by LC-MS/MS (**Figure 5** and **Table 2**). MccJ25 appeared only slightly degraded in the presence of pancreatin, highly impacted by elastase and almost unaffected in the presence of chymotrypsin (**Figure 5A**). The degradation products previously identified in the static model of duodenum digestion were searched in priority and assigned from their retention times and MS/MS spectra. Degradation products with a single hydrolysis in the Y9-I18 loop (*m/z* 709) were detected in the three conditions (**Figure 5B**). Pancreatin hydrolysis yielded mainly DP2, while elastase generated DP3 and DP4 and chymotrypsin yielded DP2 and DP3. Finally, the degradation products DP7 to DP10, corresponding to multiple hydrolysis in the loop region, were formed in the presence of pancreatin as well as in the presence of elastase (**Figures 5C–F**).

The action of pancreatic enzymes on MccJ25 was also evaluated using the antibacterial activity assay. The inhibitory activity of MccJ25 measured after each experiment by the microdilution assay is reported in **Table 3**. In the presence of trypsin, no significant decrease in Mcc25 activity was observed even after 120 min of reaction (data not shown). By contrast, pancreatin decreased the inhibitory activity of MccJ25 instantly by fourfold relative to the positive control. A further eightfold decrease then occurred over the 120 min of reaction. In the presence of elastase, the antibacterial activity was reduced instantly (since t_0_) by a factor of 256 (128 AU/mL compared to 32,768 AU/mL for the positive control, and decreased by an additional fourfold (8 AU/mL) over the next 2 h. Chymotrypsin also affected the antibacterial activity but to a lower extent, with a reduction to 2,048 and 1,024 AU/mL, respectively, at 0 and 120 min time points. These results were confirmed qualitatively by using the agar diffusion assay (**Figures 5G,H**). An inhibitory activity was detected in the presence of pancreatin at 0 min of digestion, showing an inhibition zone of 1.5 cm compared with the 1.8 cm inhibition zone of the positive control (data not shown). This inhibitory activity decreased after 120 min of digestion by pancreatin, showing an inhibition zone of 1.2 cm. Contrary to pancreatin, trypsin did not induce any reduction of the inhibitory activity of MccJ25 over digestion (data not shown). This could be explained by the absence of trypsin cleavage site in the MccJ25 sequence. The inhibitory activity of MccJ25 in presence of elastase was markedly reduced at t_0_ showing an inhibition zone of 1.1 cm compared with the positive control (1.8 cm). After 120 min of digestion, MccJ25 was shown to have totally lost its antibacterial activity as evidenced by the absence of inhibition zone. Therefore, LC-MS/MS data and antibacterial assays both indicate that MccJ25 is partially degraded in the presence of pancreatin and completely degraded in the presence of elastase after 120 min, while chymotrypsin has no significant effect on the stability and antibacterial activity of MccJ25.

## Discussion

The identification of peptide and protein degradation products in complex samples has emerged in the past years thanks to the advance in analytical techniques. Such approaches provide key information on the degradome, defined as the sum of the products of various degradation processes, which include substrates of proteases and their generated cleavage products (Strlicč et al., 2009). They rely generally on bottom-up proteomic approaches (i.e., hydrolysis followed by identification of the generated peptides through database searching) (Lai et al., 2015), and have beneficiated from biochemical methods such as N-terminal enrichment strategies (Sabino et al., 2017), gel electrophoresis-based methods: protein topography and migration analysis platform (PROTOMAP) (Dix et al., 2014; Kaisar et al., 2016) and in-gel degradomics (Vidmar et al., 2017) as well as from the development of targeted analytical workflows (Savickas and Auf dem Keller, 2017) and specific algorithms (El-Assaad et al., 2017). The [1]rotaxane lasso structure of MccJ25, yielding [2]rotaxane species upon hydrolysis, precludes the use of database search to identify degradation products. Nevertheless, the use of MS/MS-based molecular networking revealed very efficient to reveal the degradome. This study thus constitutes to our knowledge the first application of molecular networking to degradomics. In addition, the + 18 u increment permitted to assign unambiguously the hydrolysis site of the [2]rotaxane degradation products.

The present study has provided evidence of the degradation of MccJ25 in both dynamic and static models of digestion. MccJ25 was stable in the stomach compartment, but underwent multiple hydrolysis as soon as it entered the duodenal compartment, resulting in a drastic decrease in its antibacterial activity against a model *Salmonella* species. Interestingly, a slight increase of the inhibitory activity of MccJ25 was observed after 30 min of duodenal digestion followed by an additional decrease reaching the initial arbitrary activity value. This fluctuation might be due to the presence of bile salts in the duodenal medium. Bile is produced continuously by the liver and enters the duodenum via the bile duct during digestion in order to facilitate digestion by emulsifying dietary lipids (Redinger and Small, 1972). The microscopic micelles thus formed are hydrophilic at the external surface and hydrophobic inside. MccJ25 contains several hydrophobic amino acids (Rebuffat et al., 2004), which may bind to micelles formed in the duodenum and be released later, as is reported to occur in microencapsulation processes (Corona-Hernandez et al., 2013). Altogether, these data indicate that MccJ25 is degraded by proteases upon entering the duodenum. Thus, the compact and stable structure of MccJ25 permits to tolerate acidic conditions of stomach but does not provide protection toward duodenal proteases, which limit its application as orally administrated antimicrobial.

The stability of MccJ25 were later evaluated in the presence of pancreatic enzymes separately in order to determine which among the duodenal components are responsible for the MccJ25 degradation. Pancreatin is known as a mixture of five proteases, namely trypsin, chymotrypsin, elastase, carboxypeptidase A and carboxypeptidase B (Whitcomb and Lowe, 2007), the first of these having no cleavage site in the MccJ25 amino acid sequence. Carboxypeptidases A and B are exopeptidases that cleave peptide bonds at the carboxyl terminus, next to amino acids having aromatic, neutral or acidic side chains for the first one and to basic amino acids, primarily arginine and lysine, for the second (Whitcomb and Lowe, 2007). MccJ25 does not contain any cleavage site for either of these two exopeptidases. Elastase and chymotrypsin are endopeptidases that hydrolyze peptide bonds next to uncharged (Ala, Gly, Ser) and aromatic (Phe, Tyr, Trp) amino acids, respectively (Walker, 2004). Thus, MccJ25 does have cleavage sites for elastase and chymotrypsin, some of which are located in the more exposed loop region of the compact lasso structure. The action of elastase and chymotrypsin was further evaluated by LC-MS/MS. Elastase yielded the same degradation products previously detected in the presence of pancreatin (DP7–DP10) involving the F10-V11 cleavage as well as the Y9-F10 cleavage in addition to the loss of one to four residues. The action of elastase also revealed a novel degradation form DP4, not observed in the presence of pancreatin, which is located at the I13-G14 position. However, only two degraded forms with no cleaved segments resulting from the action of chymotrypsin were observed. The first degraded form is located at the Y9-F10 position was observed at the beginning of treatment with chymotrypsin but not with elastase, while the second degraded form located at the F10-V11 position was detected in the presence of both chymotrypsin and elastase. MccJ25 thus appears to have two major cleavage sites for the two pancreatic proteases: the first is located at the Y9-F10 position corresponding to the chymotrypsin cleavage site, and the second is located at the I13-G14 position which correspond to the elastase cleavage site.

The effects of pancreatic enzymes on MccJ25 were confirmed using antimicrobial activity assays. The results showed a significant decrease of the inhibitory activity of MccJ25 in the presence of pancreatin, contrary to trypsin which did not induce any significant reduction of the antibacterial activity of MccJ25 over the reaction time (data not shown). These results are consistent with the mechanism of action of trypsin, which is an endopeptidase known to participate in the digestion of proteins in the small intestine by catalyzing the hydrolysis of peptide bonds located on the carboxyl side of lysine or arginine residues (Whitcomb and Lowe, 2007), and the absence of such residues in MccJ25. A remarkable reduction of the antibacterial activity of MccJ25 was observed since the addition of elastase. This reduction was continued during the reaction time until a total loss of the activity detected at 120 min. Chymotrypsin was not shown to induce a significant effect as shown with elastase. Nevertheless, the chymotrypsin cleavage pattern was observed in samples treated with elastase. This suggests that cleavage by elastase is activated by chymotrypsin. Indeed, it has been reported that elastase is secreted by the pancreas in an inactive pro-enzyme form called proelastase and then activated in the duodenum by trypsin (Ghelis et al., 1978; Whitcomb and Lowe, 2007). This appears to explain the different degradation patterns obtained with the same enzyme according to the model.

## Conclusion

**FIGURE 6:**
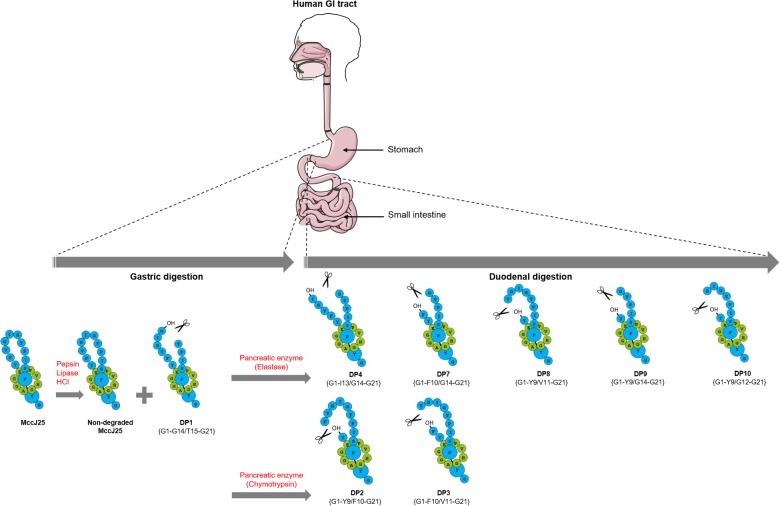
Schematic representation of MccJ25 breakdown in the human stomach and duodenum.

The present study has provided compeling evidence that despite its highly stable structure in various harsh conditions, MccJ25 is partly degraded in the human gastrointestinal tract, practically as soon as it enters the duodenum, thus losing its antibacterial activity, and that the pancreatic protease elastase is largely responsible for this degradation (**Figure 6**). This finding suggests that MccJ25 is unlikely to affect the human GI tract and may therefore be considered as a safe alternative to antibiotics and for food applications. However, this also means that in order to be considered as an antibacterial agent to be potentially used in the GI tract in man or animals, MccJ25 would have to be encapsulated or bioengineered to stabilize the loop region toward hydrolysis while maintaining the antibacterial activity.

## Author Contributions

SN participated in the experimental design and responsible for the laboratory analysis, data analysis, and writing. RH participated in the experimental design, laboratory analysis and data analysis. SZ participated in the experimental design, laboratory analysis, data analysis, and writing. BF participated in the experimental design and contributed to data analysis. SR and IF participated in the experimental design, data analysis, and writing.

## Conflict of Interest Statement

The authors declare that the research was conducted in the absence of any commercial or financial relationships that could be construed as a potential conflict of interest.

## Footnotes

1 http://gnps.ucsd.edu
