# Supplementary material for: Fate and Biological Activity of the Antimicrobial Lasso Peptide Microcin J25 Under Gastrointestinal Tract Conditions
Source: Front Microbiol. 2018 Aug 3;9:1764. doi: 10.3389/fmicb.2018.01764 (PMC6085462; doi:10.3389/fmicb.2018.01764)
Supplement: Supplementary file 1 [file Presentation_1.PDF]

Supplementary Material

Fate and biological activity of the antimicrobial lasso peptide microcin J25 under gastrointestinal tract conditions

Sabrine Naimi, Séverine Zirah, Riadh Hammami, Benoît Fernandez, Sylvie Rebuffat and Ismail Fliss*

*** Correspondence:** Corresponding Author: ismail.fliss@fsaa.ulaval.ca

1 Supplementary Figures and Tables

1.1 Supplementary Table

Table S1. Degradation pattern of MccJ25 in the *in vitro* digestive models. Incubation time of first detection (min), experimental and calculated monoisotopic *m/z*, corresponding error (ppm), molecular mass and retention times (RT) of the species detected on the molecular network for MccJ25 and its degradation products.

Inc. time (min)	Ion species	Exp. *m/z*	Calc. *m/z*	Error (ppm)	M (Da)	RT (min)	Assignment	Degradation product
0	$[M+3H]^{3+}$	703.0145	703.0143	-0.57	2106.02	8.6	MccJ25	-
	$[M+2H]^{2+}$	1054.0172	1054.0178	-0.28				
	$[2M+3H]^{3+}$	1405.0187	1405.0213	-1.85				
	$[M+2H+Na]^{3+}$	710.3404	710.3416	-1.69				
	$[M+3H-H_2O]^{3+}$	697.0103	697.0108	-0.72				
	$[M+H+NH_4]^{2+}$	1062.5296	1062.5311	-1.41				
	$[M+H+Na]^{2+}$	1065.0087	1065.0088	-0.09				
**Stomach dynamic model (TIM-1)**								
60	$[M+3H]^{3+}$	709.0184	709.0178	0.85	2124.03	7.2	{G1-G14/T15-G21}	DP1
	$[M+2H]^{2+}$	1063.0231	1063.0231	0.00				
**Duodenum static model**								
0	$[M+3H]^{3+}$	709.0182	709.0178	0.56	2124.03	7.0	{G1-Y9/F10-G21}	DP2
	$[M+2H]^{2+}$	1063.0229	1063.0231	-0.19				
0	$[M+3H]^{3+}$	709.0186	709.0178	1.13	2124.03	7.3	{G1-F10/V11-G21}	DP3
	$[M+2H]^{2+}$	1063.0236	1063.0231	0.47				
0	$[M+3H]^{3+}$	709.0184	709.0178	0.85	2124.03	7.4	{G1-I13/G14-G21}	DP4
	$[M+2H]^{2+}$	1063.0234	1063.0231	0.28				
60	$[M+3H]^{3+}$	671.3238	671.3231	1.04	2010.95	6.6	{G1-G12/G14-G21}	DP5
60	$[M+3H]^{3+}$	652.3175	652.3160	2.30	1953.93	6.9	{G1-V11/G14-G21}	DP6
60	$[M+3H]^{3+}$	619.2935	619.2932	0.48	1854.86	6.6	{G1-F10/G14-G21}	DP7
60	$[M+3H]^{3+}$	659.9955	659.9950	0.76	1976.96	6.5	{G1-Y9/V11-G21}	DP8
	$[M+2H]^{2+}$	989.4889	989.4889	0.00				
60	$[M+3H]^{3+}$	570.2706	570.2704	0.35	1707.79	5.7	{G1-Y9/G14-G21}	DP9
	$[M+2H]^{2+}$	854.9018	854.9019	-0.12				
120	$[M+2H]^{2+}$	939.9551	939.9547	0.43	1877.89	6.2	{G1-Y9/G12-G21}	DP10

1.2 Supplementary Figures

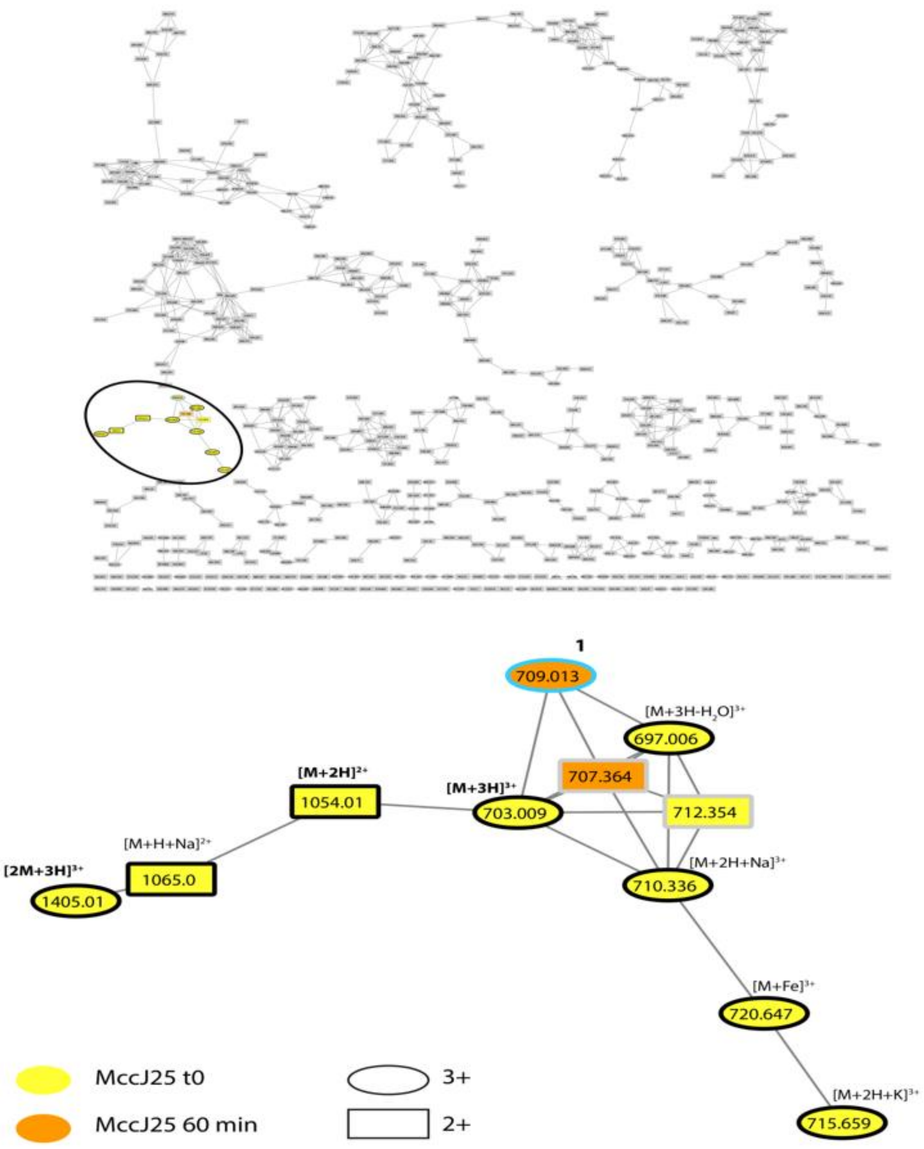

Figure S1. Molecular network showing MccJ25 degradome in the stomach compartment of the TIM-1 dynamic model of digestion. The cluster corresponding to MccJ25 is enlarged. The nodes assigned to MccJ25 are bordered in black and annotated. The node corresponding to hydrolysed MccJ25 is bordered in blue and numbered as in Table 2. The nodes not considered to delineate the degradome are bordered in grey. They correspond to mixed precursor selection in MS/MS experiments.

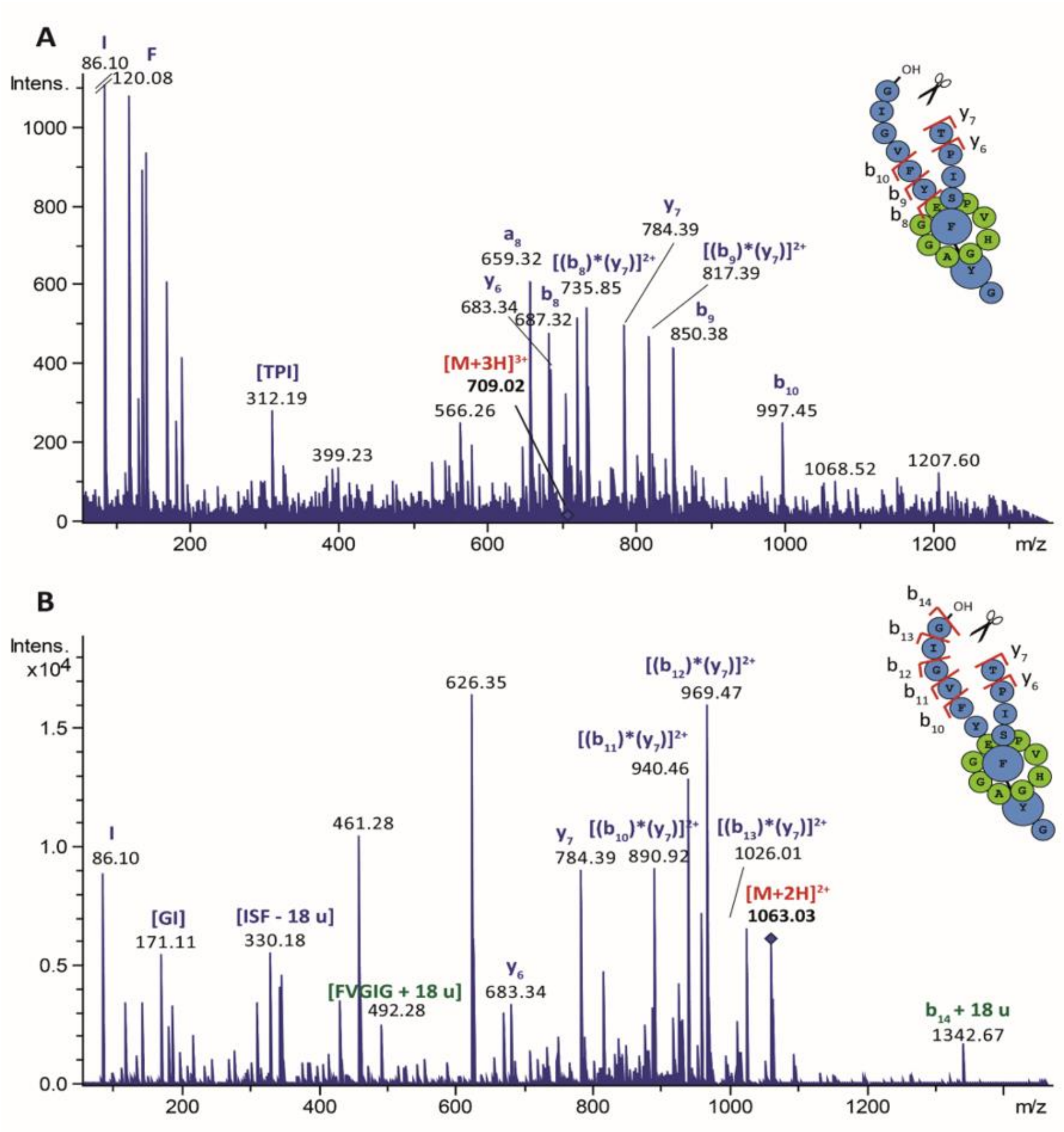

Figure S2. MS/MS spectra of MccJ25 hydrolyzed at G14-T15 formed in the stomach compartment of the TIM-1 dynamic model, DP1 {G1-G14/T15-G21}. A: $[M+3H]^{3+}$ (*m/z* 709.02, CE 32.6 eV), B: $[M+2H]^{2+}$ (*m/z* 1063.03, CE 40 eV). The hydrolysis site was determined from the + 18 u increment product ions (in green).

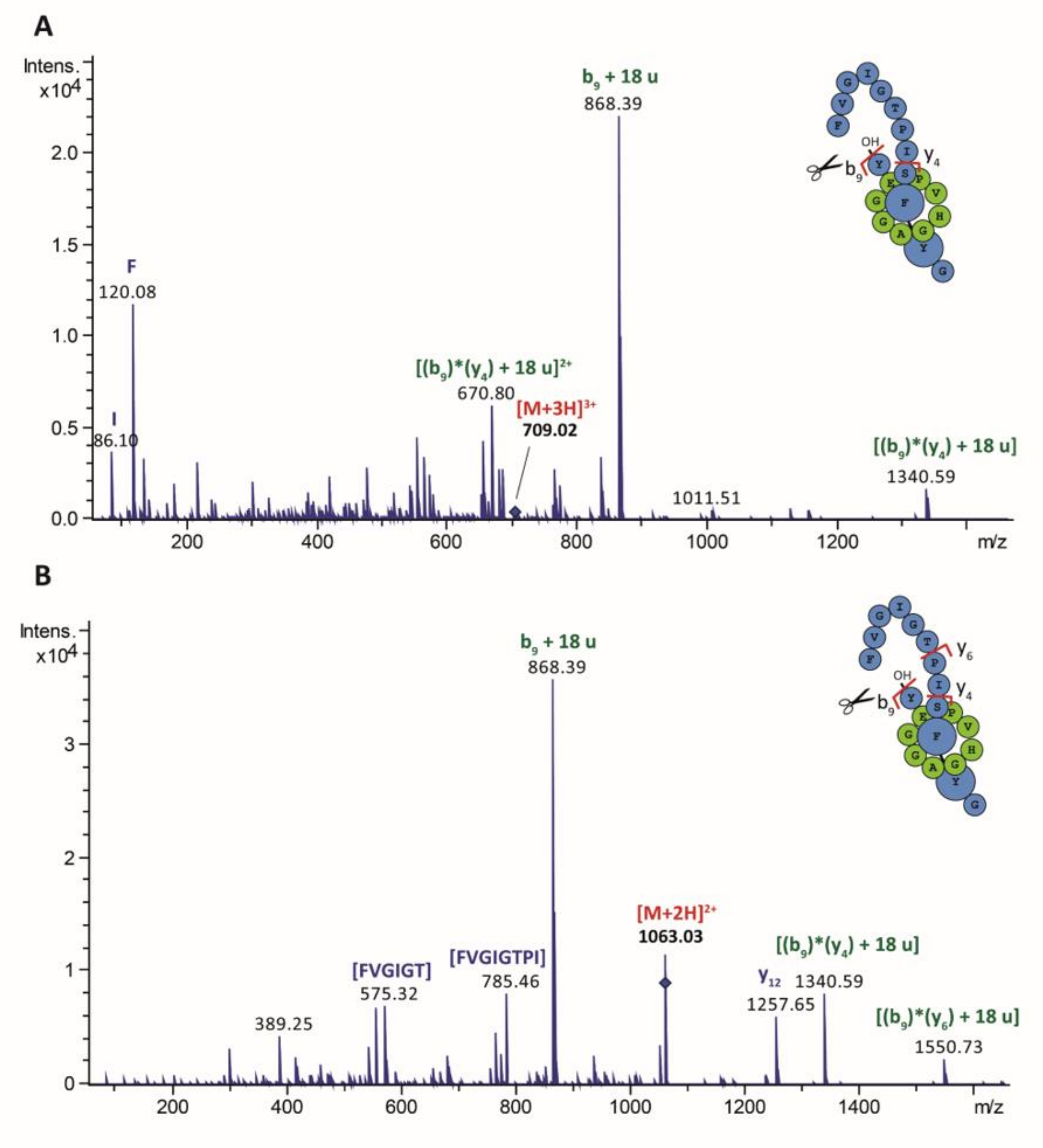

Figure S3. MS/MS spectra of MccJ25 hydrolyzed at Y9-F10 formed in the static model of duodenum, DP2 {G1-Y9/F10-G21}. A: $[M+3H]^{3+}$ (*m/z* 709.02, CE 32.6 eV), B: $[M+2H]^{2+}$ (*m/z* 1063.03, CE 40 eV). The hydrolysis site was determined from the + 18 u increment product ions (in green).

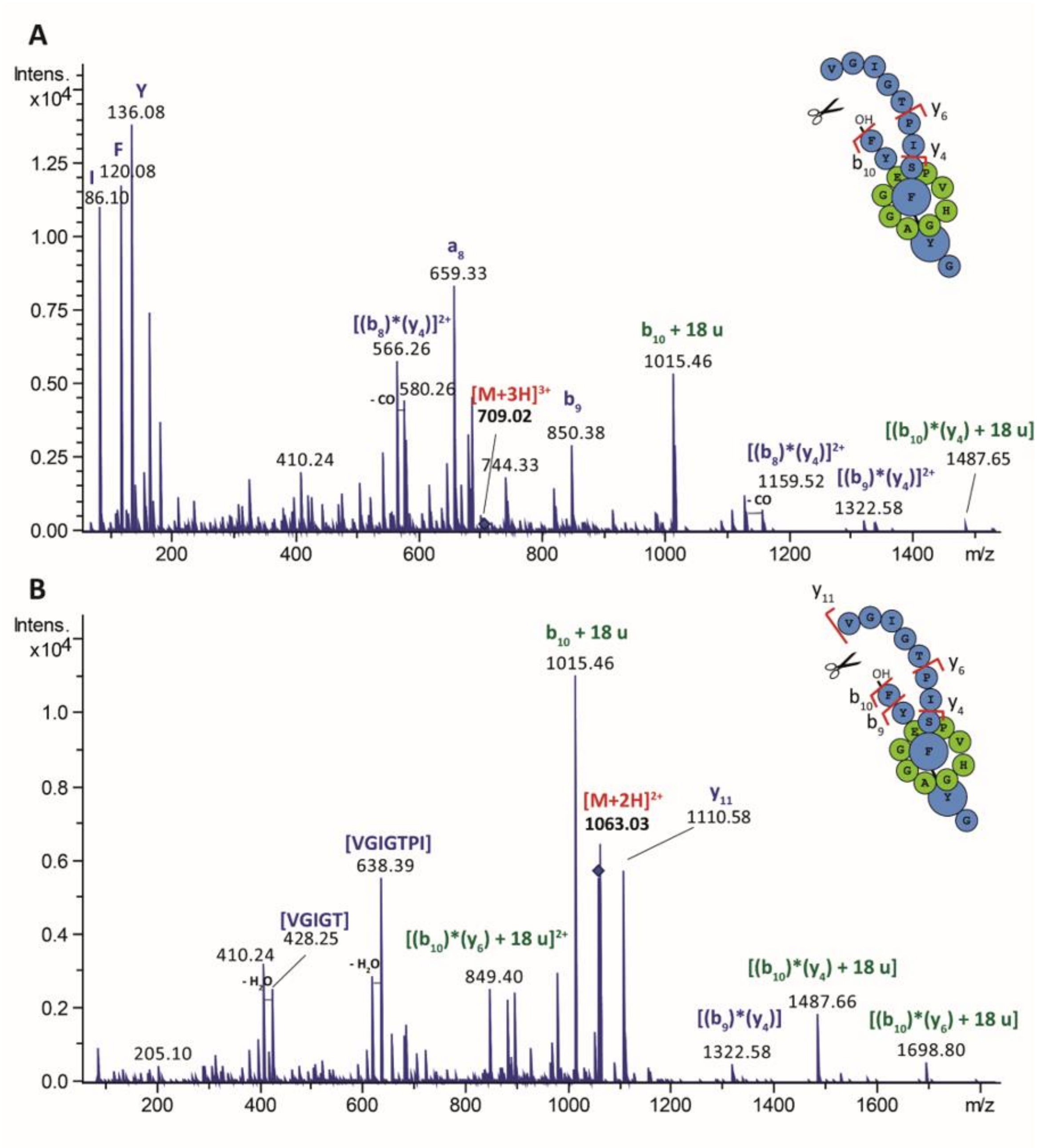

Figure S4. MS/MS spectra of MccJ25 hydrolyzed at F10-V11 formed in the static model of duodenum, DP3 {G1-F10/V11-G21}. A: $[M+3H]^{3+}$ (*m/z* 709.02, CE 32.6 eV), B: $[M+2H]^{2+}$ (*m/z* 1063.03, CE 40 eV). The hydrolysis site was determined from the + 18 u increment product ions (in green).

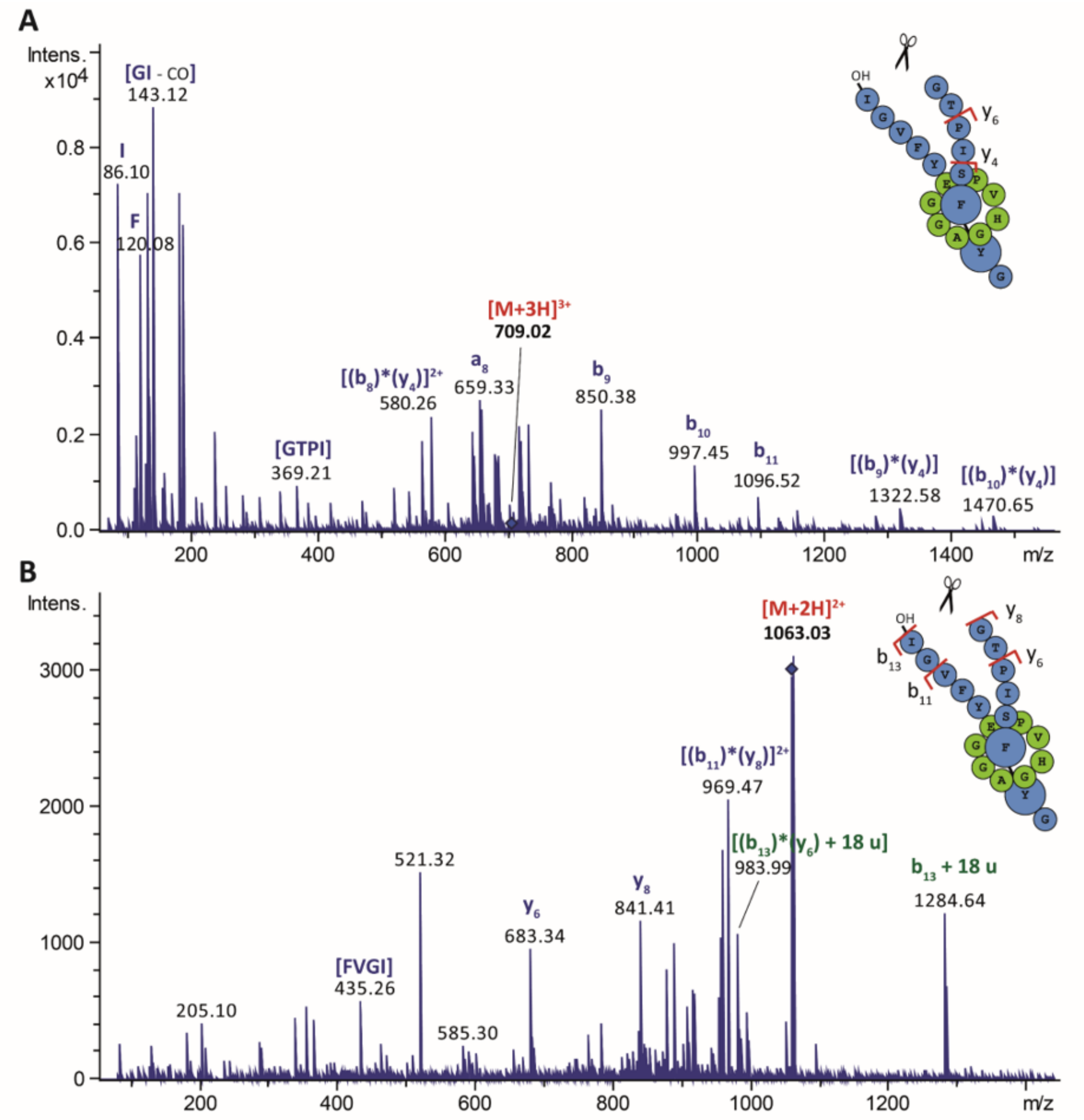

Figure S5. MS/MS spectra of MccJ25 hydrolyzed at I13-G14 formed in the static model of duodenum, DP4 {G1-I13/G14-G21}. A: $[M+3H]^{3+}$ (*m/z* 709.02, CE 32.6 eV), B: $[M+2H]^{2+}$ (*m/z* 1063.03, CE 40 eV). The hydrolysis site was determined from the + 18 u increment product ions (in green).

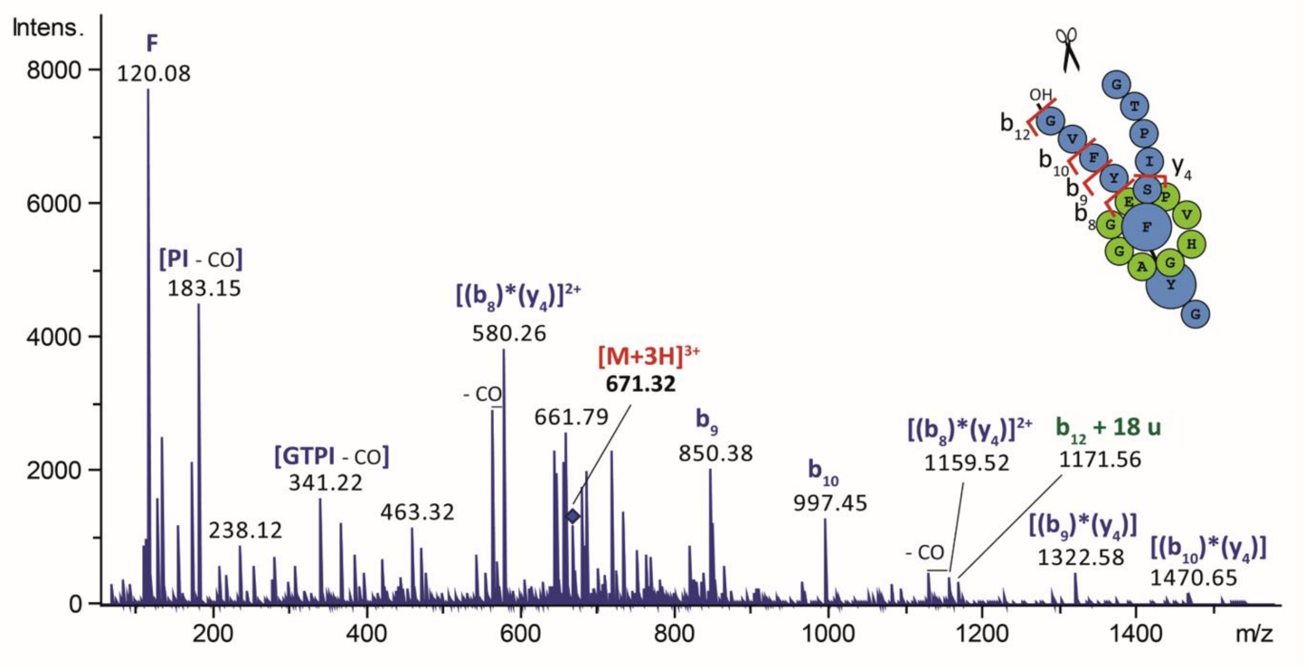

Figure S6. MS/MS spectra of MccJ25 hydrolyzed both at G12-I13 and I13-G14 formed in the static model of duodenum, DP5 {G1-G12/G14-G21}: $[M+3H]^{3+}$ (*m/z* 671.32, CE 30.3 eV). The hydrolysis site was determined from the + 18 u increment product ions (in green).

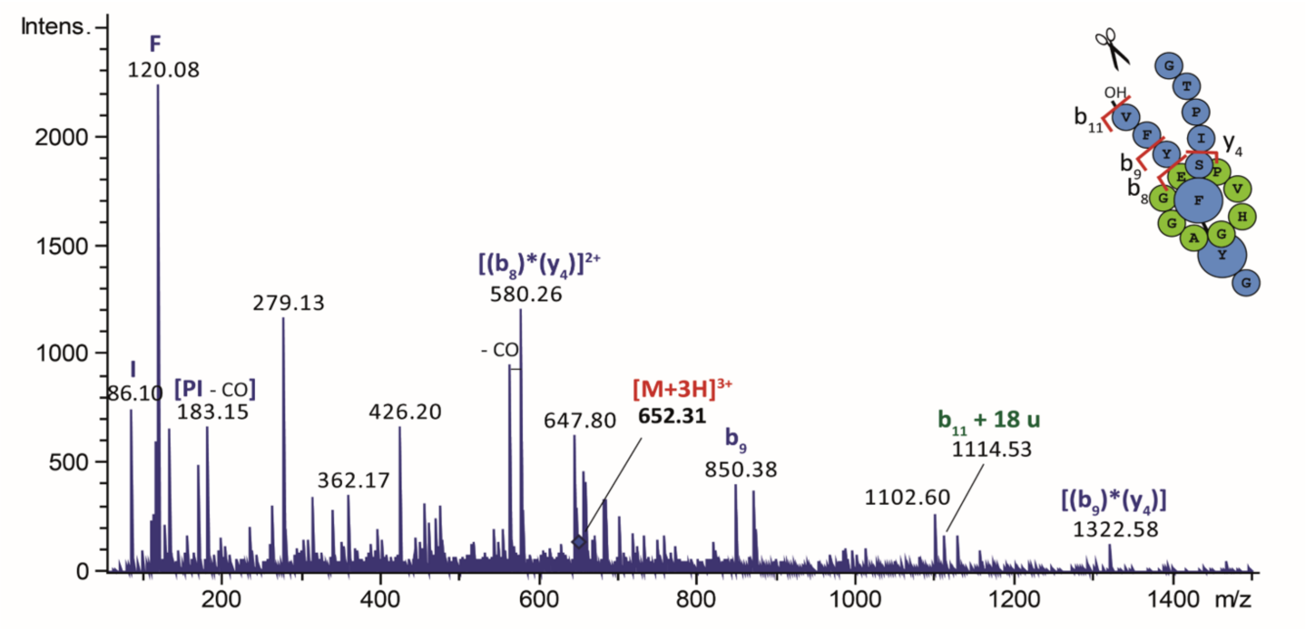

Figure S7. MS/MS spectra of MccJ25 hydrolyzed both at V11-G12 and I13-G14 formed in the static model of duodenum, DP6 {G1-V11/G14-G21}: $[M+3H]^{3+}$ (*m/z* 652.31, CE 29.2 eV). The hydrolysis site was determined from the + 18 u increment product ions (in green).

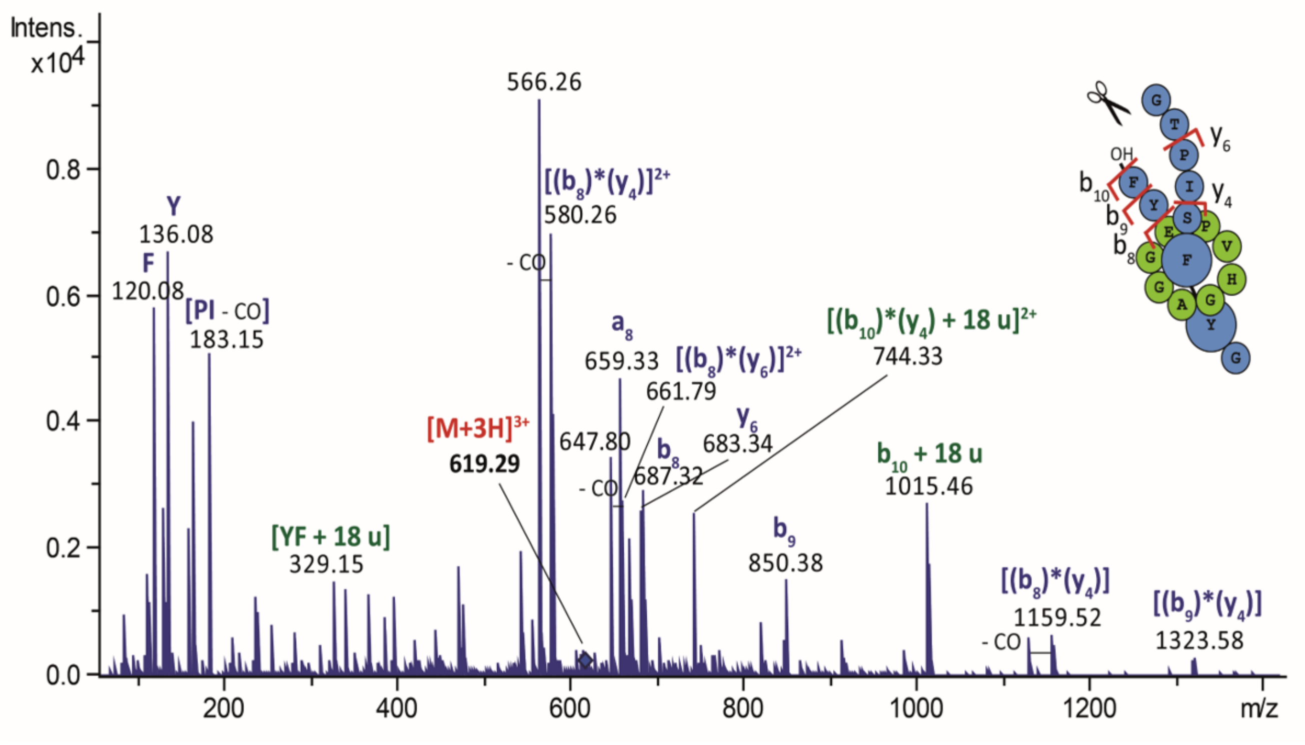

Figure S8. MS/MS spectra of MccJ25 hydrolyzed both at F10-V11 and I13-G14 formed in the static model of duodenum, DP7 {G1-F10/G14-G21}: $[M+3H]^{3+}$ (*m/z* 619.29, CE 27.2 eV). The hydrolysis site was determined from the + 18 u increment product ions (in green).

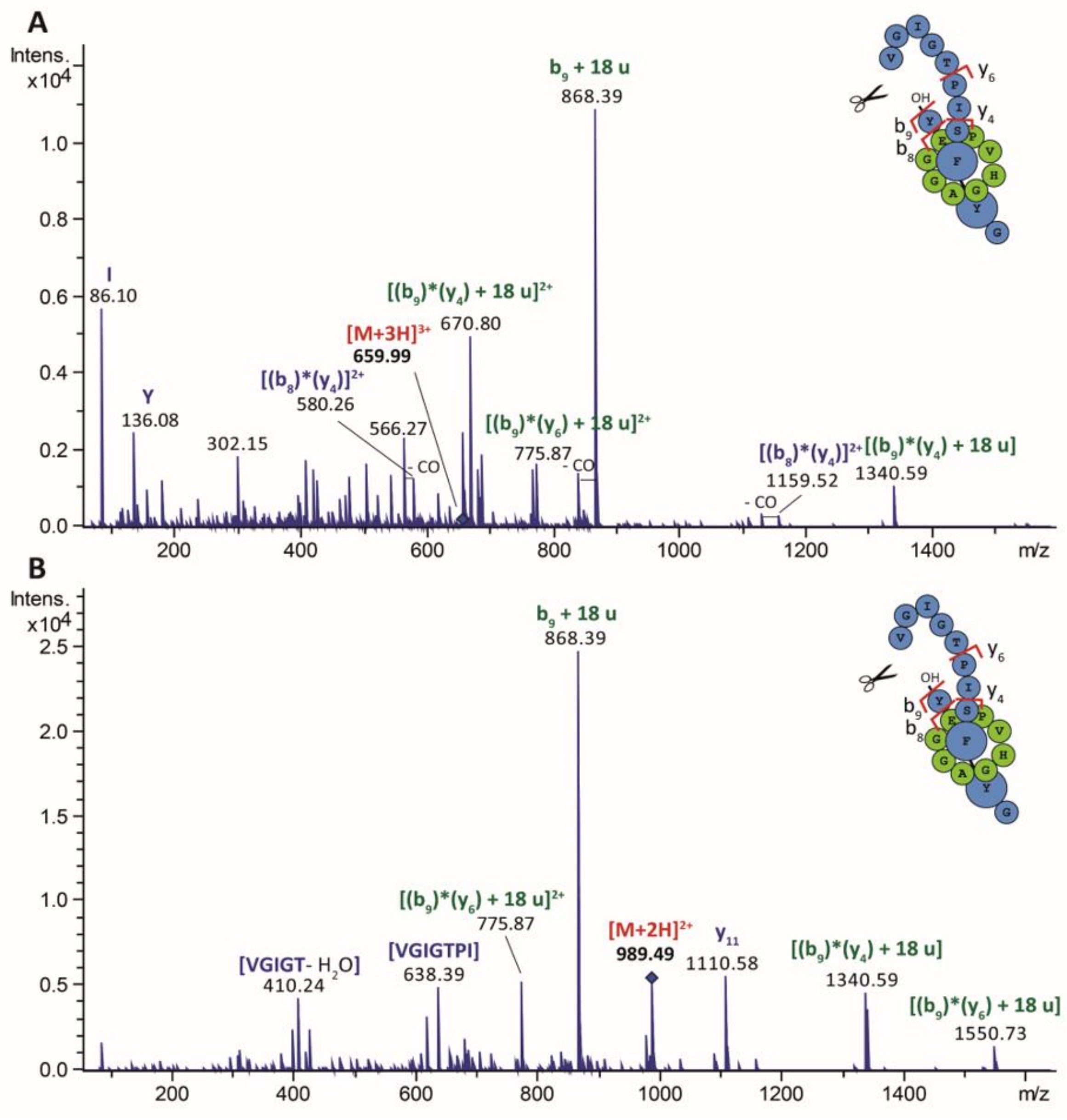

Figure S9. MS/MS spectra of MccJ25 hydrolyzed after at Y9-F10 and F10-V11 formed in the static model of duodenum, DP8 {G1-Y9/V11-G21}. A: $[M+3H]^{3+}$ (*m/z* 659.99, CE 29.6 eV), B: $[M+2H]^{2+}$ (*m/z* 989.49, CE 39.8 eV). The hydrolysis site was determined from the + 18 u increment product ions (in green).

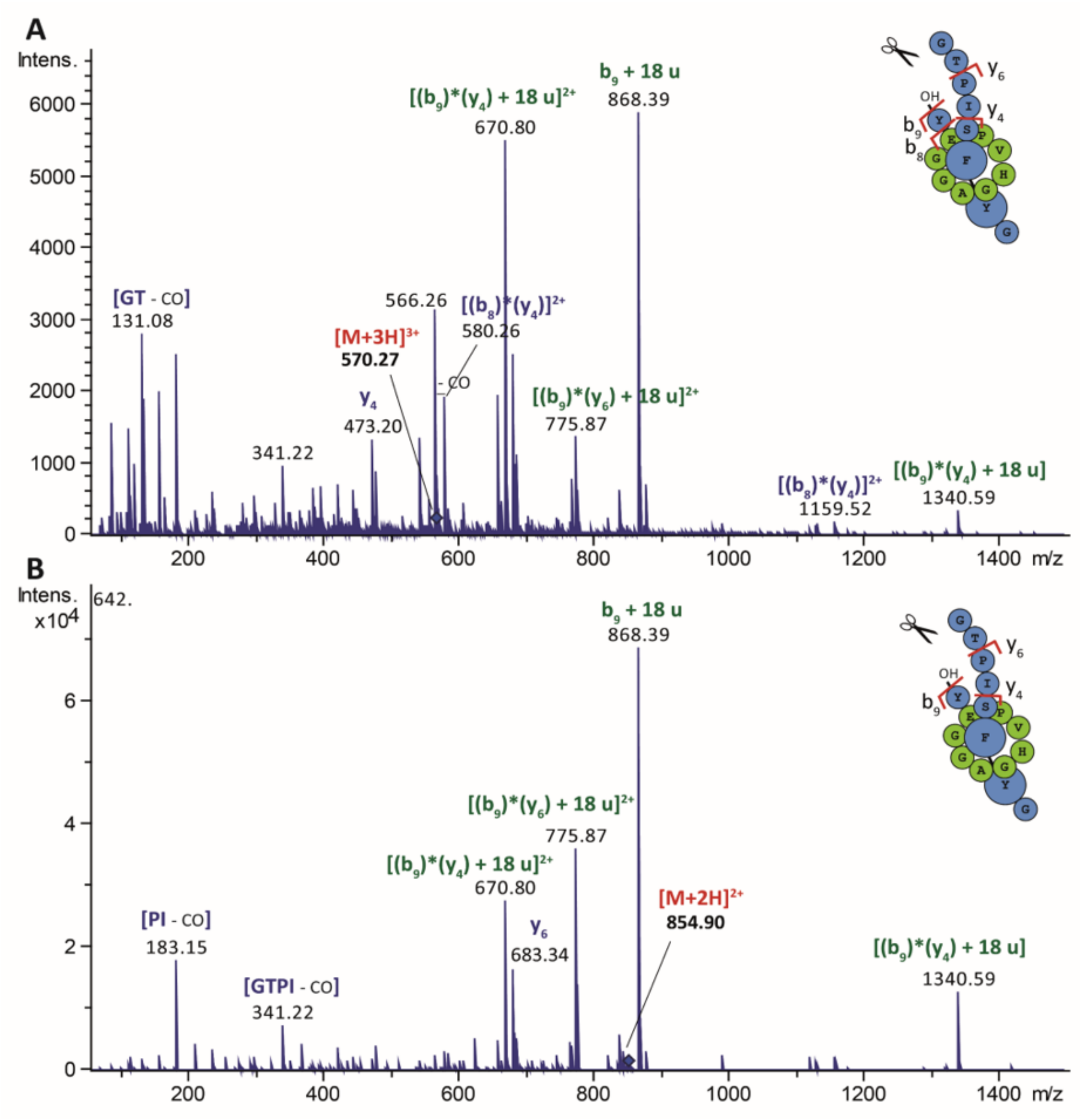

Figure S10. MS/MS spectra of MccJ25 hydrolyzed both at Y9-F10 and I13-G14 formed in the static model of duodenum, DP9 {G1-Y9/G14-G21}. A: $[M+3H]^{3+}$ (*m/z* 570.27, CE 24.2 eV), B: $[M+2H]^{2+}$ (*m/z* 854.90, CE 37.1 eV). The hydrolysis site was determined from the + 18 u increment product ions (in green).

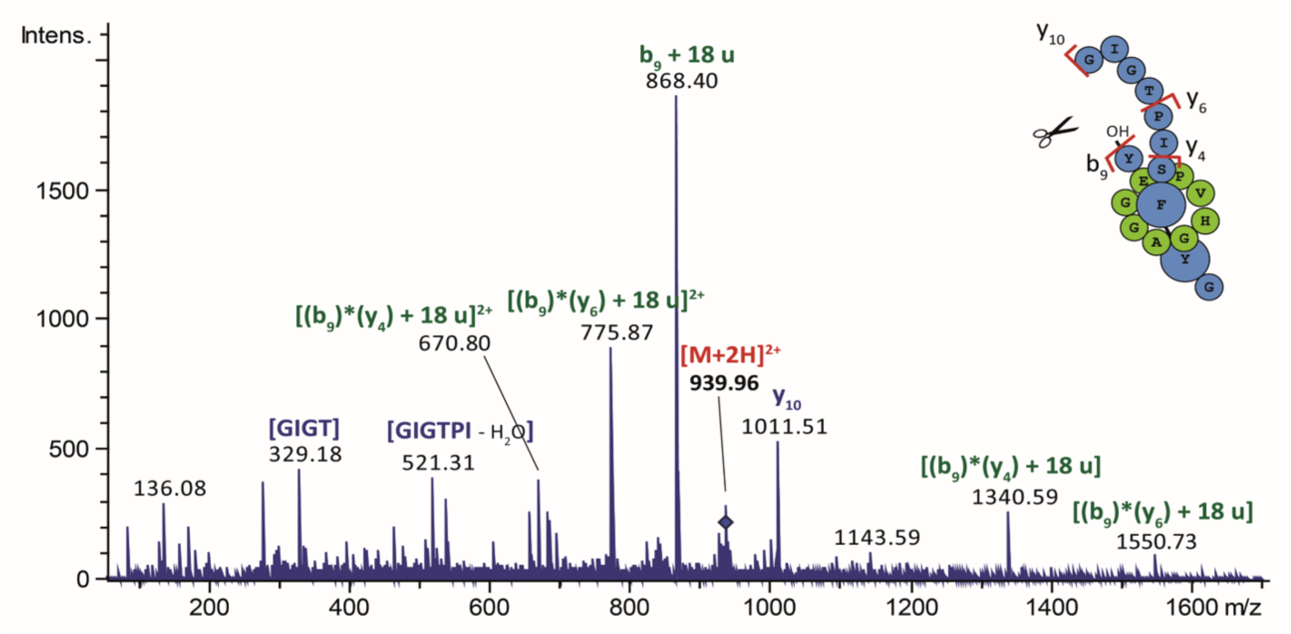

Figure S11. MS/MS spectra of MccJ25 hydrolyzed both at Y9-F10 and V11-G12 formed in the static model of duodenum, DP10 {G1-Y9/G12-G21}: [M+2H]$^{2+}$ (*m/z* 939.96, CE 38.8 eV). The hydrolysis site was determined from the + 18 u increment product ions (in green).
